# Retrograde Vital Pulp Treatment in External Root Resorption Due to Third Molar Impaction: A Proof-of-Concept and Case Report

**DOI:** 10.3390/jcm14165828

**Published:** 2025-08-18

**Authors:** Emanuele Ambu, José Luis Sanz, Roberto Ghiretti, Francesco Bellucci, Carlo Gaeta, Simone Grandini, James Ghilotti, Leopoldo Forner

**Affiliations:** 1 Unit of Endodontics, Department of Medical Biotechnologies, Periodontology, Restorative and Paediatric Dentistry, University of Siena, 53100 Siena, Italy; leleambu@libero.it (E.A.); odontoiatriagaeta@libero.it (C.G.); grandini@unisi.it (S.G.); 2 Departament d’Estomatologia, Facultat de Medicina I Odontologia, Universitat de València, 46010 Valencia, Spain; james.ghilotti@uv.es (J.G.); forner@uv.es (L.F.); 3 Private Practitioner, 46047 Porto Mantovano, Italy; roberto.ghiretti@libero.it; 4 Private Practitioner, 83100 Avellino, Italy; infostudiobellucci@gmail.com

**Keywords:** retrograde obturation, vital pulp treatment, case reports, root resorption, impacted teeth

## Abstract

**Background/Aim**: Third molar impaction with the consequent root resorption of second molars often creates complexities in treatment planning and execution. In the past, the root canal treatment (RCT) of second molars was required in these cases to avoid pulp necrosis and infection. The aim of this paper is to report a surgical/retrograde approach for the maintenance of pulp vitality, proposed as retrograde vital pulp treatment (rVPT), in cases of asymptomatic or reversibly affected teeth with root resorptions caused by impacted adjacent teeth. **Methods**: A case report on the rVPT of two upper second molars with root resorption due to third molar impaction is presented. The chief complaint of the patient was a slight pain during bite involving the upper second molars. Heat and cold sensitivity tests were performed, suggesting a healthy pulp status. A cone beam computed tomography (CBCT) scan was performed to aid the diagnosis and treatment planning, showing bilateral upper third molar impaction and both distal roots of the upper second molars affected by external root resorption (ERR). In both cases, the third molar was surgically extracted, the surface of the root with ERR was smoothened and rVPT was carried out by performing a 3 mm retrograde preparation of the root canal and its retrograde sealing using a hydraulic calcium silicate-based cement (hCSCs). **Results:** Heat and cold sensitivity tests were performed 1 month, 3 months, 6 months and 1 year after the treatment. The patient reported no pain, and the pulp sensitivity was maintained in all follow-up periods. A CBCT scan was performed 24 months after the treatment, reporting a complete perirradicular endogenous bone apposition. **Conclusions**: Based on the successful clinical and radiographic outcomes observed in the present case after two years of follow-up, rVPT is proposed for the maintenance of pulp vitality in cases of asymptomatic or reversibly affected teeth with ERR caused by impacted adjacent teeth.

## 1. Introduction

The dentin–pulp complex is exposed to numerous potentially harmful factors. Most commonly, caries and dental trauma pose a risk to the integrity of the cellular component and tissues that comprise its structure [1,2]. This complex possesses a series of defence mechanisms, which are encompassed in the concept of tertiary dentinogenesis [3]. Tertiary dentinogenesis comprises the production of mineralized tissue at the dentin–pulp interface in response to a harmful stimulus. This process involves the differentiation of dental pulp stem cells or DPSCs towards a neo-odontoblastic lineage for their acquisition of the ability to synthesise and secrete a tertiary dentin matrix [4,5].

This physiological response is the basis for vital pulp treatment (VPT). VPT aims to maintain the tooth’s vitality upon a stimulus that causes a histologically reversible damage to the dentin–pulp complex by providing a biocompatible environment that allows and, ideally, enhances local tissue repair and regeneration [6]. This approach comprises a series of procedures which involve the removal of infected and/or irreversibly damaged tissue and the disinfection and capping of the remaining affected tissue with biocompatible and bioactive materials which favour its re-mineralization [7,8].

VPT has shown high success rates in clinical trials on patients with clinical signs and symptoms of varying degrees of pulpitis, both using the traditional (reversible/irreversible) [9,10,11] and Wolter’s diagnostic classification (mild/moderate/severe) [12,13]. This success has been attributed to the introduction of pulp cappers with favourable biological properties, among other factors [14]. Specifically, calcium silicate-based cements (CSCs) are recommended in evidence-based protocols and statements as the materials of choice in this regard [8,15].

CSCs are categorized as biocompatible and bioactive materials [16]. The latter refers to their ability to form hydroxyapatite on their surface and induce a mineral attachment to the dentin substrate through an ionic interchange with the surrounding tissue fluids [17]. Additionally, numerous studies on diverse lineages of dental stem cells (DSCs) have demonstrated their ability to induce their osteo/odonto/cementogenic differentiation and mineralization [18,19]. As a result, CSCs can also be used in different endodontic procedures, i.e., as apical plugs in apexification procedures, as coronal barriers in regenerative endodontic treatment, as root-end filling materials in microsurgical endodontics, and as root repair materials [20].

The advances in VPT for the management of carious and traumatic lesions and the extensive supporting evidence in this regard [21,22] may raise the question of its potential application in other relevant clinical situations, such as ERRs caused by impacted adjacent teeth. This pathology may exhibit an inflammatory response of varying degrees or remain asymptomatic [23]. Additionally, they may develop dental caries, further root resorptions and/or periodontal changes if untreated [24].

Traditionally, root resorptions involving the periodontium, cementum, dentin and pulp were managed via root canal treatment (RCT), placing an apical plug in the affected root [25]. Currently, there is a lack of reliable evidence regarding their management. Thus, treatment planning is case-dependent and aims to manage the cause of the resorption and aid the survival of the tooth with root resorption [26]. In this regard, the aim of this paper is to describe a surgical/retrograde approach for the maintenance of tooth vitality, proposed as retrograde vital pulp treatment (rVPT), in cases of asymptomatic or reversibly affected teeth with ERR caused by impacted adjacent teeth.

## 2. Case Report

Firstly, the patient was verbally informed about the novel but evidence-based nature of the procedure to be performed, which ultimately aimed to maintain the vitality of the affected second molars. The possibility of pulp necrosis after the treatment was highlighted, as well as the indication for RCT if this was the case. Then, a written informed consent form was obtained after the patient understood and gave approval for the surgical extraction of the upper impacted third molars and retrograde vital treatment (rVPT) of the upper second molars with root resorption.

The present manuscript was written in accordance with the Preferred Reporting Items for Case reports in Endodontics (PRICE) 2020 guidelines [27].

### 2.1. Patient History and Chief Complaint

The patient was a 27-year-old Caucasian male with the following chief complaint: discomfort and mild pain when biting on both upper second molars. The patient’s dental and medical history, as well as his family history, were non-contributory. The chronogram of the patient’s diagnosis, treatment and follow-up is illustrated in Table 1.

### 2.2. Diagnosis

The following clinical tests were performed: palpation, percussion, inspection, periodontal probing and heat sensitivity tests. Palpation did not cause pain, while percussion caused mild to moderate pain in both upper second molars. Clinical inspection did not reveal any gingival inflammation, which appeared healthy. Periodontal probing exhibited probing depths of less than 3 mm. No carious lesions were observed. Heat (using heated gutta-percha) and cold sensitivity tests (using Endo Cool spray; Cyber Tech, Northampton, UK) were performed bilaterally on the upper first and second molars, as well as the second premolars, both exhibiting normal responses. Based on these findings, the preoperative diagnosis was a normal pulp (positive response to pulp sensitivity tests) exposed to a harmful mechanical stress (molar impaction).

Complementarily, the patient provided a CBCT scan, which had been performed using WhiteFox (Acteon, Lyon, France) (voxel 150 microns, 8 × 8 cm FOV), and standard settings (25 s of exposure time, 90 kV and 7 mA). The images were viewed and interpreted by an Endodontist with 25 years of experience and by a maxillo-facial specialist with more than 30 years of experience. The examination of the CBCT scan revealed that both upper third molars were impacted and mesio-inclined, and their crowns had caused a severe resorption of the distal roots of both upper second molars, both reaching their respective root canals.

### 2.3. Treatment Plan and Execution

The treatment of the affected molars was divided into two visits, one for the upper right quadrant and one for the upper left quadrant, 1 month apart (Table 1). The same treatment protocol was performed in both visits, as follows: anesthesia was administered using one vial of Articaine 1:100,000 and two vials of Lidocaine 1:50,000 to increase the hemostatic effect. A trapezoidal flap was then elevated, and an osteotomy of the buccal bone was performed to access the third molar and allow its extraction.

For the second part of the procedure, we propose the term retrograde vital pulp treatment (rVPT) for the treatment of vital teeth with ERR involving cementum, dentin and pulp.

This conservative approach involves three phases:

(1) The smoothening of the ERR to allow for a clear visualization and access to the pulp exposure.
(2) The creation of a retrograde cavity and partial pulpotomy of the pulp exposure.
(3) The placement of a pulp capping agent in the cavity over the remaining vital pulp tissue.

Following this treatment plan, the distal root of the second molar with ERR was slightly trimmed using a sterile diamond bur, smoothening the surface. The surface was examined using an operative microscope (Extaro 300; Zeiss, Oberkochen, Germany) and micro-mirrors (MM4 and MM5; Hu-Friedy, Chicago, IL, USA), revealing the root canal pulp tissue. A retrograde cavity was performed using 3 mm retrotips (AS 3—Satelec; Acteon, Mérignac, France), irrigated with an abundant flow of water, and then a retrograde filling was placed with a hCSC (Biodentine; Septodont, Saint-Maur-des-Fosses, France) inserted with a micro-compactor (PLGBK6; Hu-Friedy, Chicago, IL, USA). The hCSCs were left to set for 15 min and were then polished and shaped using a sterile Arkansas stone dental bur (Shofu Dental Corporation, San Marcos, CA, USA). The surgical flap was then repositioned and sutured with 5-0 sutures (Braun Surgical, Rubi, Barcelona, Spain).

A post-operative limited CBCT scan was performed (Kavo OP3D, KaVo, Biberach, Germany) (voxel size 125 micron, 5 × 5 limited field of view) using standard exposure (95 kV, time exposure 13 s 9 mA). The CBCT was examined using Cliniview 11 software (Instrumentarium Dental; Tuusula, Finland).

### 2.4. Follow-Up

At follow-up appointments, the treated molars were asymptomatic and functional. No recurrence of pain was reported by the patient during the follow-up period. Heat (using hot gutta-percha) and cold (using Endo Cool spray, Cyber Tech) sensitivity tests were performed at 1-, 3-, 6- and 12-month post-treatment, showing normal pulp responses in both upper second molars. A CBCT scan (Kavo OP3D, voxel size 125 micron, 5 × 5 limited field of view) using standard exposure (95 kV, time exposure 13 s 9 mA) was performed two years after the treatment, showing radiographic evidence of no further resorption of the roots, and healthy perirradicular bone surrounding both the upper second molars.

**Figure 1:**
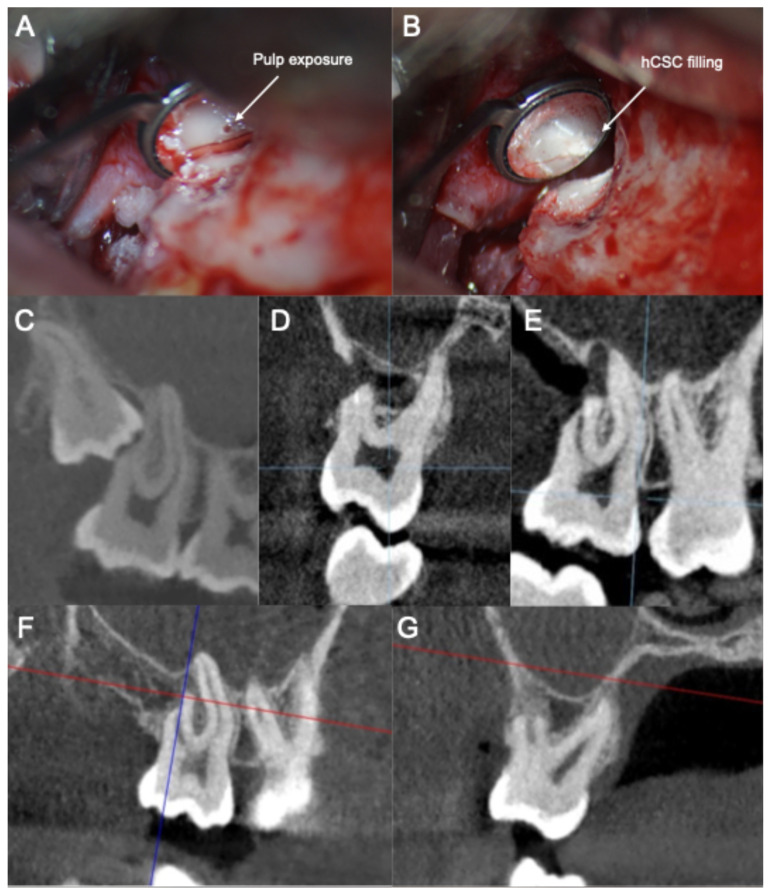
Retrograde vital pulp treatment of the distal root of the upper right second molar due to the impaction of the upper right third molar. (**A**) Trimmed surface of the distal root of the second molar after the extraction of the third molar. (**B**) Retrograde pulp capping with a calcium silicate-based cement. (**C**) Impacted third molar and the resorption of the distal root of the second molar (preoperatory). (**D**,**E**) Post-operatory three-dimensional sealing of the pulp exposure (sagittal view (**D**); coronal view (**E**)). (**F**,**G**) Presence of osseous tissue neoformation surrounding the distal root of the second molar after 2 years of follow-up (coronal view (**F**); sagittal view (**G**)).

**Figure 2:**
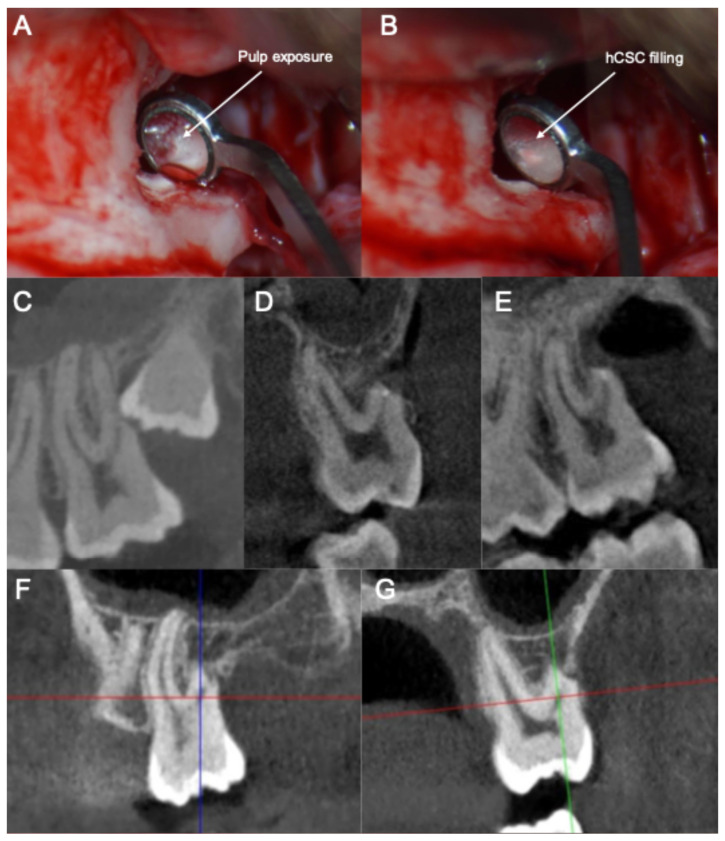
Clinical and radiographic documentation of the retrograde vital pulp treatment of the distal root of the upper left second molar due to the impaction of the upper left third molar. (**A**) Trimmed surface of the distal root of the second molar after the extraction of the third molar. (**B**) Retrograde pulp capping with a calcium silicate-based cement. (**C**) Impacted third molar and the resorption of the distal root of the second molar (preoperatory). (**D**,**E**) Post-operatory three-dimensional sealing of the pulp exposure (sagittal view (**D**); coronal view (**E**)). (**F**,**G**) Presence of osseous tissue neoformation surrounding the distal root of the second molar after 2 years of follow-up (coronal view (**F**); sagittal view (**G**)).

The clinical and radiographic documentation of the clinical cases is depicted in Figure 1 and Figure 2. Images were acquired as complementary diagnostic tools and were evaluated by the clinicians in the different treatment phases. They are included in the present manuscript to illustrate the written case description.

## 3. Discussion

ERR is the formation of defects in the cementum and dentin of the root surface [23]. Several factors, such as chronic periodontitis, pressure from an impacted tooth, tumors, trauma and orthodontic treatment, can cause ERR through the activation of odontoclasts and cementoclasts, involving multiple molecular interactions. The mesio-inclination and horizontal inclination of the third molar have been suggested to increase the risk of ERR on the distal aspect of the adjacent second molar [28]. Dao and colleagues reported that resorptions caused by impacted teeth were 8.8%; the prevalence of impacted tooth resorption was highest for molars at 14.9%, compared to 0% for premolars and 6.4% for anterior teeth [29]. This resorption can be classified into four grades, from grade 1 (no resorption or limited to cementum) to grade 4 (severe resorption with exposure of the pulp) [30].

Some studies suggest no treatment for asymptomatic second molars after the surgical extraction of the third molar, with only follow-up and observation, mainly in younger patients. For example, the study by Qu and colleagues reported that the asymptomatic ERR of second molars has a high probability of maintaining vitality without further intervention after third molar extraction, recommending only long-term observation. However, 11% of second molars showed loss of vitality, requiring endodontic treatment. The direct exposure of the pulp to the oral environment may adversely affect its vitality. In the same study, pulp necrosis was associated with older patients (>35 years old), likely due to root canal calcification and vascular sclerosis. In cases of symptomatic second molars, endodontic treatment before the third molar extraction is suggested, performing an apical plug in the presence of an open apex [23]. Currently, clinical guidelines from major endodontic societies stress the priority of conservative and/or minimally invasive endodontic procedures, shifting towards regenerative and biologically based treatments [8,15,31]. Thus, the RCT of a tooth with an ERR with a healthy or reversibly affected pulp status may be considered unnecessary or overtreatment if more conservative alternatives are available. Additionally, the potential intraoperative and postoperative complications of RCT [32] would be avoided.

In the two cases reported in the present study, the patient complained of mild pain when biting on the upper second molars. After proposing different treatment options (extraction of the third molar without further treatment, endodontic treatment before extraction, rVPT of the root with ERR), the patient chose rVPT. Based on current evidence regarding conventional VPT, teeth eligible for this treatment should exhibit a normal pulp status or reversible pulpitis (following the traditional classification) or the equivalent using Wolters’ classification [12]. Nevertheless, cases should be individualized to consider potential factors that may influence the outcome of the treatment, i.e., age, underlying pathologies, etc. [33,34]. The application of rVPT in cases with irreversible pulpitis is currently discouraged, based on the lack of evidence in this regard. In case of complications, i.e., irreversible pulpitis, pulp necrosis, sealing failure and/or infection, an RCT could be performed.

Despite the growing amount of literature addressing the use of hCSCs for VPT, studies reporting the use of these materials for retrograde treatment in the absence of orthograde endodontic treatment are still rare, if not essentially non-existent. An interesting paper discusses the possibility of vital root resection in severely furcation-involved maxillary molars after full pulpotomy using hCSCs, demonstrating the potential of these materials in maintaining the vitality of the root canal pulp [35].

To our knowledge, the only article that describes a comparable surgical procedure was performed by Dongzhe and colleagues [36]. They describe a case involving the extraction of an impacted lower left third molar and a similar microsurgical approach to maintain the vitality of the distal root of the second molar, with a 1-year follow-up and a successful outcome. In multi-rooted teeth, when part of the root is removed, although some of the apical blood supply is lost, the pulp can continue to remain vital due to compensation from blood flow coming from the other roots, provided the cavity is thoroughly cleaned and sealed promptly [37]. An additional article suggests the use of hCSCs to maintain the vital pulp of the entire remaining tooth after the complete removal of the fractured mesial root of a lower first molar. The authors suggest that the use of hCSCs, along with an operating microscope and microsurgical instruments, aids the maintenance of the vital pulp, reducing extraction and the risk of fracture associated with traditional therapies [38].

The reported cases confirm the possibility of maintaining pulp vitality in second molars with severe ERR due to third molar impaction, even in the presence of pain. The possibility of performing rVPT is strictly connected to the clinician’s skills in using endodontic microsurgery instruments, as well as the position of the root with ERR. The limited access to roots positioned more palatally may hinder the procedure. Thus, the use of a CBCT scan is paramount for treatment planning. As reported by Hermann and colleagues, CBCT can change the treatment plan in 58.6% of cases that initially received planning using panoramic radiographs [39].

This case report presents several limitations. First, only two cases were presented; furthermore, both treatments were performed on the same patient. The uncommon nature of the presented cases, along with the demand for advanced clinical skills and specific instruments, limits their availability. Nevertheless, the novelty of the proposed treatment procedure, the presence of evidence to justify its biological basis, and the reported successful outcome after 2 years of follow-up justify the consideration of the present study as a proof of concept, which may serve as a base for future clinical studies.

## 4. Conclusions

Based on the successful clinical and radiographic outcomes reported in the present study after two years of follow-up, rVPT is proposed as a viable approach for the maintenance of tooth vitality in cases of asymptomatic or reversibly affected teeth with root resorptions caused by impacted adjacent teeth. Following the present proof-of-concept, further clinical studies with larger sample sizes and follow-up periods are necessary to confirm the reported results.

## Figures and Tables

**Table 1:** Diagnosis, treatment and follow-up chronogram.

Visit	Diagnosis/Treatment	Observations
Initial visit	Anamnesis, clinical and radiographic examination (CBCT)	Mesio-inclined impacted upper third molars with resorption of the distal root of the adjacent second molars.
	Extraction of the right third molar and rVPT of distal root of the upper right second molar	No significant complications.
Second visit (1 month)	Extraction of the left third molar and rVPT of distal root of the upper left second molar	No significant complications.
1-, 3-, 6- and 12-month follow-up	Heat and cold sensitivity tests	Pulp responding normally. Patient is asymptomatic.
24-month follow-up	Heat and cold sensitivity tests; CBCT scan	Pulp responding normally. Patient is asymptomatic. Periadicular endogenous bone apposition observed in the CBCT.

## Data Availability

The data are not publicly available due to privacy concerns, but may be made available from the corresponding author upon reasonable request and with permission from the patient.
